# Protection motivation theory and smoking quitting intention: findings based on structural equation modelling and mediation analysis

**DOI:** 10.1186/s12889-022-13263-0

**Published:** 2022-04-27

**Authors:** Haoxiang Lin, Meijun Chen, Qingping Yun, Lanchao Zhang, Chun Chang

**Affiliations:** Department of Social Medicine and Health Education, School of Public Health, Peking University Health Science Center, 38 Xueyuan Rd, Haidian District, Beijing, China

**Keywords:** Protection motivation theory, Quitting intention, Smoking

## Abstract

**Objective:**

Although many smoking cessation strategies have been implemented, only a few strategies at the population level are grounded in theory. Even in those interventions based on specific theories, most studies have focused only on the outcome. The main objective of this study was to demonstrate the utility of protection motivation theory (PMT) in explaining smoking quitting behaviour among adults, with the goal of providing valuable evidence for further intervention strategies.

**Method:**

This was a cross-sectional study. Participants were randomly selected on the street from 26 provinces in mainland China. Data were collected via face-to-face interviews. Cronbach’s alpha coefficient and the interclass correlation coefficient (ICC) were used to assess the reliability of the individual PMT constructs. We applied structural equation modelling (SEM) to test how well the PMT constructs predicted intention. A bootstrap test was performed to test the potential mediators.

**Results:**

The Cronbach’s alpha coefficients of all the subscales ranged from 0.71 to 0.74. Greater intentions were significantly associated with higher threat appraisal (Coef. = 0.18, *P* < 0.01) and coping appraisal (Coef. = 0.24, *P* < 0.01). Threat appraisal was significantly associated with higher perceived severity and vulnerability but inversely associated with extrinsic rewards and intrinsic rewards. Coping appraisal was significantly associated with higher self-efficacy and response efficacy but inversely associated with response cost. The R^2^ of quitting intention was 0.12, which means that 12% of quitting intention was predicted by PMT constructs. For threat appraisal, approximately 19.8% of the effects on lower threat appraisal were mediated by higher extrinsic rewards. For coping appraisal, approximately 42.8% of the effects on higher coping appraisal were mediated by higher response efficacy.

**Conclusion:**

This study finds that PMT is a sound theoretical framework for predicting smoking quitting intention among adults. Coping appraisal has a stronger effect than threat appraisal for predicting quitting intention. Mediation analyses confirmed that extrinsic rewards and response efficacy mediated the relationship between PMT constructs and quitting intention. Our findings are essential for understanding quitting behaviour among adults and support more effective smoking cessation activities.

## Introduction

Smoking is one of the leading preventable causes of death and is predicted to claim 200 million lives in China this century, predominantly among the poorest and most vulnerable people [1, 2]. Although many smoking cessation strategies have been implemented, only a few strategies at the population level are grounded in or guided by specific theories [3, 4]. A large number of studies have shown the importance of incorporating theory-guided interventions in smoking cessation programmes [5–7]. Therefore, integrating theories of behaviour change into routine smoking cessation strategies has become increasingly important for both scholars and policy makers in all countries. Furthermore, even in these interventions based on specific theories, most studies have focused only on the outcome (e.g., whether quitting intention enhances smoking reduction). There are unanswered questions. For example, what happens in the quitting process? How do these influencing factors interact with each other? Are there any potential mediators? These questions are still understudied and have implications for other studies.

Until recently, the association between different factors related to smoking behaviours has been investigated by a few studies [8–11]. For example, a study found that reducing second-hand smoke exposure (SHS) is not simply the result of the implementation of smoke-free workplace policies. Rather, the change may reflect a synergism of multiple factors, with such forces working in conjunction to contribute to lower SHS exposure [8]. Another study confirmed that a smoke-free workplace policy can not only reduce SHS exposure but also have spill-over effects on smoking by controlling drinking behaviour. However, the topics related to quitting intention are almost untouched by researchers [11].

Protection motivation theory (PMT) is a well-known Western theory of behaviour change and is especially useful for addressing health behaviour. A common feature of such models is the assumption that components are causally related. In the case of PMT, it is assumed that environmental and intrapersonal factors lead to an appraisal of threat and an appraisal of coping and that these appraisals lead to protection motivation, which may in turn lead to more or less adaptive behaviours [12, 13].

**Fig. 1:**
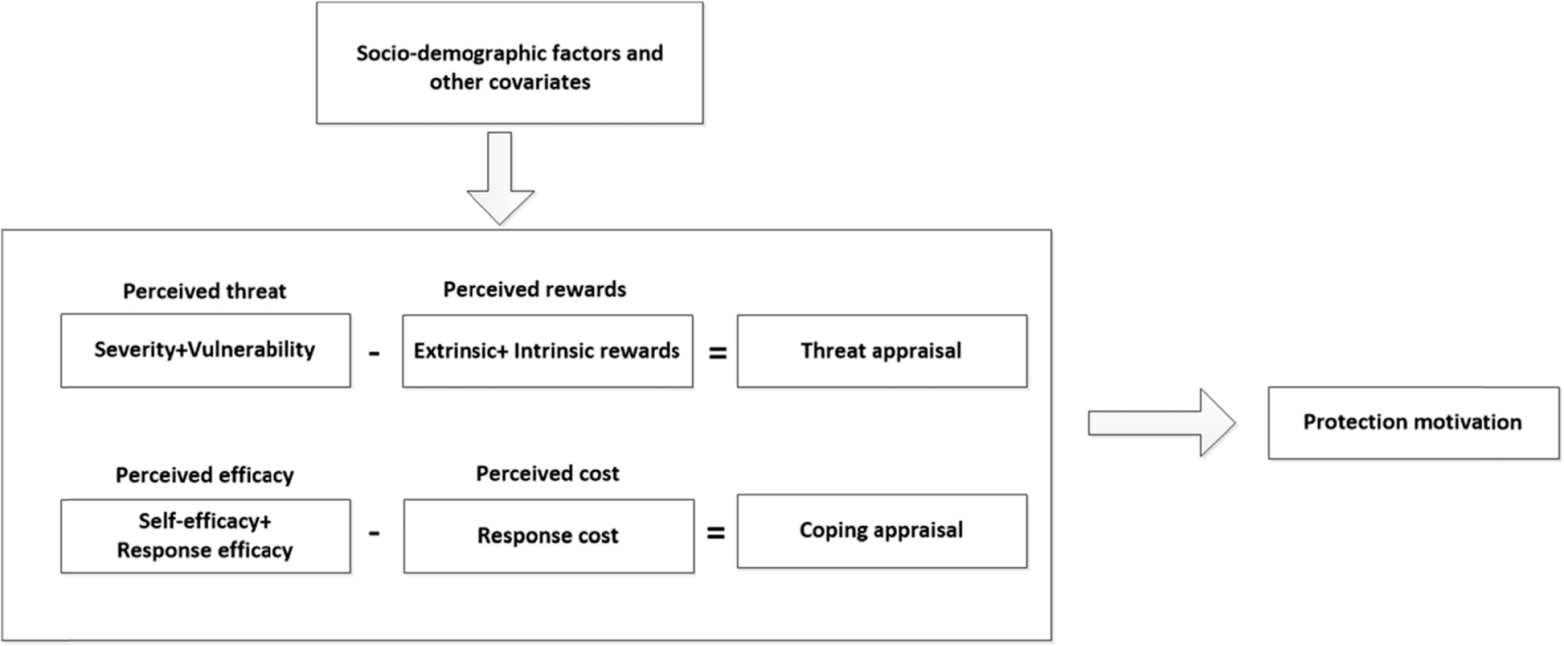
Protection motivation theory framework

Specifically, PMT suggests that a person’s ‘protection motivation’ is determined by two processes. As shown in Fig. 1, threat appraisal is one dimension and serves as an evaluation of maladaptive behaviours. It is determined by an individual's beliefs about the negative consequences of the health threat (perceived severity), his or her vulnerability to the negative consequences of the threatened event (perceived susceptibility) and the benefits of the performance of the maladaptive behaviour (intrinsic and extrinsic rewards). Greater motivation for specific health behaviours (such as quitting intention) can be expected if the perceived severity and vulnerability are high and rewards are low [14].

Coping appraisal is also a potential pathway for ‘protection motivation’ and serves as an evaluation of a person’s ability to manage and avoid the threat. It is determined by an individual's beliefs about the effectiveness of the preventative behaviour for the threatened event (response efficacy), confidence in one's ability to perform the preventative behaviour (self-efficacy) and barriers to performance of the preventative behaviour (response costs). Response efficacy and self-efficacy are expected to enhance coping appraisal, whereas response costs are expected to reduce coping appraisal [15, 16].

In the area of smoking, PMT has received some attention in predicting smoking behaviour. For example, Thrul et al. showed that coping appraisal and practical self-efficacy were predictors of smoking behaviour [17]. Yan et al. found that perceived rewards from smoking and self-efficacy of not smoking reduced their intention to and actual use of tobacco [4]. Another study found that copping appraisal had much stronger effects than threat appraisal when used for the prevention of smoking [18]. However, thus far, little research has used PMT to examine quitting intention among adults. We focus on quitting intention for two reasons. First, according to the 2015 China Adult Tobacco Survey, the majority of smokers had no quitting intention (only 17.6% of smokers wanted to quit smoking within a year). Currently, limited resources can be used in smoking cessation in China; therefore, a study of how to trigger strong quitting intention in routine tobacco control activities is needed. Second, we recognize that actual quitting behaviour is important. However, most smokers will experience repeated relapse, and it is difficult or even impossible to measure the final effects on behaviour in a study; therefore, we believe that quitting intentions are more important than short-term quitting attempts, as they ‘get ball rolling’ in the long term.

In addition, the internal process and how these influencing factors interact with each other have all been ignored. Studying the association between PMT constructs has potential public health implications, as it demonstrates the possible channel to maximize the intervention effect. For example, our previous study found that alcohol drinking is among the channels that mediate the association between smoking prevalence and SHS exposure in workplaces. Therefore, it is suggested that the objective of reducing SHS exposure can be achieved through smoke-free policies. However, in terms of triggering stronger quitting intentions, companies should integrate multilevel health promotion, such as alcohol drinking intervention programmes [11].

There are also other theories used currently in the field of smoking behaviour. The most commonly used is the transtheoretical model of change (TTM). It is a systematic theory that states that health behaviour is determined on a stage­to­stage basis and divides behaviour into five stages: precontemplation, contemplation, preparation, action and maintenance. For any stage, specific interventions should be provided to strengthen the behaviour change occurrence and/or support to achieve the next stage [12, 13]. However, the measurement of the stage is sometimes not easy and equivocal; therefore, it is difficult to identify the most effective intervention [19].

The theory of planned behaviour (TPB) is another important behaviour change theory that proposes that behavioural intentions are a function of attitudes and subjective norms. The theory adds the construct of perceived behavioural control as an additional predictor of intention and behaviour [13]. A meta-analysis of 17 studies found that although the TPB has been proven to be an effective theoretical framework for designing health behaviour interventions, the reciprocal link between intervention techniques and TPB variables targeted remains unclear [20].

The main objective of this study was to demonstrate the utility of PMT in explaining quitting intention among adults, with the goal of providing valuable evidence for further smoking cessation strategies. Specifically, we propose the following hypotheses. (1) PMT is a sound theoretical framework for predicting quitting intention among adults. (2) Not all PMT measures have the same strength in predicting quitting intention. (3) There are potential mediators of the pathway. These forces work in conjunction to influence quitting intention.

## Methods

### Procedure

All study procedures were approved by the ethics commission of Peking University Health Science Centre (Ethical approval number: IRB00001052-18,055). This was a cross-sectional study conducted from July to August 2020. The School of Public Health, Peking University Health Science Centre sent investigators to 26 provinces in all. Our study covers the majority of 31 provinces in mainland China, except Shanghai, Tianjin, Xinjiang, Tibet and Qinghai. Participants were randomly selected on the street in large business districts and residential areas in urban regions. Data were collected via face-to-face interviews. The survey took for approximately 8–10 min for each participant to complete. We did not provide any incentives for participation.

### Participants

We recruited participants who met the following criteria: (1) were aged between 18 and 60 years old; (2) were daily smokers, with ≥ 1 year of smoking duration; (3) smoked cigarettes; (4) lived in the current location for ≥ 5 years; and (5) agreed to participate in the survey.

### Sample size

The sample size calculation was based on a cross-sectional design. According to the 2018 China Adult Tobacco Survey [21], only 5.6% of smokers had quitting intentions, so we assumed that 6.0% of the population would have quitting intentions; a value of 0.80 (beta = 0.20) was used for power, and 0.05 was used for alpha. Therefore, the minimum sample size was 550.

### Data collection and informed consent

A four-stage method was applied in this survey. In the first stage, experts at Peking University designed the standardized questionnaires. In the second stage, an online survey system was developed, and a specific internet link was generated. In the third stage, the interviewers introduced the content and purpose of the survey according to specific introductions to potential participants and obtained verbal informed consent from all participants. All the participants were informed that the statistical analyses would be conducted anonymously and that their information would be used for research purposes and published. At the last stage, all the investigators input the information through the internet after they completed the face-to-face interviews.

The online survey system had a self-check function and can automatically identify missing data, logical errors and illegal characters.

### Measurements

#### The measurements of PMT constructs

PMT constructs were assessed using the PMT scale, which was based on the work of Xu et al. [14]. We also improved and adjusted some of the questions to make them fit for measures of quitting intention. Validity and reliability tests of the questionnaire were conducted and have been reported elsewhere [15]. Specifically, the scale comprised 21 items and used a 7-point Likert-type response scale from 1 (definitely disagree) to 7 (definitely agree). Each construct subscale included three items, and we computed the mean as the subscale score.

Perceived severity was measured by three items: “The earlier a person starts smoking, the greater the harm”, “More smokers get sick than nonsmokers”, and “Smokers die earlier than nonsmokers”. Perceived vulnerability was measured by three items: “I would become addicted if I smoked”, “I would get sick if I smoked”, and “If I smoked, I may die earlier”. Intrinsic rewards were measured by three items: “Smoking makes people feel comfortable”, “Smoking helps people concentrate”, and “Smoking enhances brainwork”. Extrinsic rewards were measured by three items: “Smokers look cool and fashionable”, “Smoking is good for social networking”, and “The life of a smoker is happier than that of a nonsmoker”. Self-efficacy was measured by three items: “I am confident that I can quit smoking successfully”, “I have the ability to stop smoking”, and “I think stop smoking is easy for me”. Response efficacy was measured by three items: “People will feel good by not smoking”, “People will be less likely to get disease if they do not smoke”, and “Quitting smoking is good for disease recovery”. Response cost was measured by three items: “A person may be isolated if he or she quits smoking”, “Refusing a cigarette offer is very impolite”, and “One will miss the enjoyment if he or she quits smoking”.

#### Measurements of threat appraisal and coping appraisal

We created two PMT pathway scores based on the seven PMT constructs described above. The threat appraisal pathway was computed as the difference between perceived threat (computed as the mean of vulnerability and severity items) and perceived rewards (computed as the mean of extrinsic rewards and intrinsic rewards items). The coping appraisal pathway was computed as the difference between the perceived efficacy (computed as the mean of response efficacy and self-efficacy items) and perceived cost (equal to the response cost construct score).

#### Measurements of quitting intention

Intention to quit smoking was assessed with two questions: “I plan to stop smoking in the next month” and “I expect to stop smoking in the next month”. We also used a 7-point Likert-type response scale from 1 (definitely disagree) to 7 (definitely agree). This has been used by other researchers [16]. We computed the mean as the subscale score.

#### Other variables

We also collected several variables of individual characteristics, such as sociodemographic information, including gender, age, BMI, marital status, ethnicity, education, yearly income, chronic diseases, and job. Smoking status, including the number of cigarettes per day, smoking duration, withdrawal symptoms, smoking harm awareness and e-cigarette usage, was assessed in the second section of the questionnaire. Smoking cessation information, including the questions ‘Have you ever tried to quit smoking?; ‘What was the longest time you quit smoking?’; and ‘When would you like to quit smoking now?’ in the third section of the questionnaire.

### Data analysis

Our data analysis was conducted in three steps. First, we assessed the reliability of the individual PMT constructs. Second, we tested how well the PMT constructs predicted quitting intention. Then, we performed a mediation analysis to assess possible mediators. SPSS 19.0 was used for the reliability test, and AMOS 24.0 was used for structural equation modelling (SEM) and mediation analysis.

#### Step 1: Assess the reliability of the individual PMT constructs

Cronbach’s alpha coefficient and the interclass correlation coefficient (ICC) were used to assess the reliability of the individual PMT constructs. A Cronbach’s alpha coefficient > 0.7 indicated good internal consistency [18].

#### Step 2: Test how well the PMT constructs predict quitting intention

We applied SEM to test how well the PMT constructs predicted quitting intention. SEM was a better method for our study than correlation or multiple regression analyses because it could be used to test overall models rather than individual coefficients and because it incorporates multiple dependent and mediating variables [22, 23].

We used the following model fit statistics that have proven to be meaningful in SEM [23, 24]: Bentler’s comparative fit index (CFI): recommended > 0.90; Tucker–Lewis index (TLI): recommended > 0.90; normed fit index (NFI): recommended > 0.9; root mean square error of approximation (RMSEA): recommended ≤ 0.08.

#### Step 3: Mediation analysis

We then performed a bootstrap test to estimate how much of the influence of the PMT constructs on threat appraisal and coping appraisal was affected by various mediators. This method is widely used to evaluate whether a factor mediates a relationship between two variables [25, 26]. In addition, as a supplemental method, we also tested the mediating effects using a Sobel test [25]. As the result was almost the same, we report only the bootstrap test results herein.

## Results

### Descriptive statistics

In total, 738 smokers were approached. After screening, 613 were identified to be eligible for analyses. Table 1 shows the descriptive statistics of the overall sample. A majority (53.2%) of the respondents were under 39 years old, with a mean (standard deviation) age of 37.95 ± 14.31 years. A total of 58.7% of respondents had a college or higher educational level. Most smokers reported smoking fewer than 100 cigarettes per week. Approximately forty-eight percent of them intended to quit (i.e., they stated a desire to quit within 6 months).

**Table 1:** Descriptive statistics in the overall sample

Demographics	n/%
**Age**	
18–29	240 (39.2)
30–39	86 (14.0)
40–49	133 (21.7)
50 and above	154 (25.1)
Mean (SD)	37.95 (14.31)
**Sex**	
Male	562 (91.7)
Female	51 (8.3)
**Ethnicity**	
Han	544 (88.7)
Others	69 (11.3)
**Marriage**	
Single	230 (37.5)
Married	359 (58.6)
Divorced or widowed	24 (3.9)
**Education attainment**	
High school/lower	253 (41.3)
College/above	360 (58.7)
**Number of cigarettes per week**	
1–50	287 (46.8)
51–100	125 (20.4)
101–150	163 (26.6)
> 150	38 (6.2)
Mean (SD)	74.86 (90.48)
**Have quitting intention**	
Yes	297 (48.5)
No	316 (51.5)
**Have chronic disease**	
Yes	104 (17.0)
No	509 (83.0)
**Total**	613

### Reliability of the individual PMT constructs

The mean scores, Cronbach’s alpha coefficients and ICCs of the PMT constructs are shown in Table 2. The mean score (SD) of the 21 items ranged from 6.19 (1.48) for Item 1 to 2.75 (1.82) for Item 10. The Cronbach’s alpha coefficient of the 21 items was 0.71. The Cronbach’s alpha coefficients of all the subscales ranged from 0.73 to 0.79. Therefore, these measures showed good internal consistency.

**Table 2:** Item response and reliability of the PMT scale

Perceived appraisal	Item and Primary Subconstructs	Mean(SD)	ICC	Cronbach α
**Perceived threat**	**Perceived severity**			0.79
	1. The earlier a person starts smoking, the greater the harm	6.19(1.48)	0.31	
	2. More smokers get sickness than nonsmokers	5.24(1.97)	0.45	
	3. Smokers die earlier than nonsmokers	5.02(1.96)	0.48	
	**Perceived vulnerability**			
	4. I would become addicted if I smoked	5.17(2.08)	0.14	
	5. I would get sick if I smoked	4.88(1.90)	0.47	
	6. If I smoked, I may die earlier	4.50(1.99)	0.44	
**Perceived rewards**	**Intrinsic rewards**			0.76
	7. Smoking makes people feel comfortable	5.04(1.81)	0.16	
	8. Smoking helps people concentrate	4.92(1.79)	0.22	
	9. Smoking enhances brainwork	5.04(1.79)	0.22	
	**Extrinsic rewards**			
	10. Smokers look cool and fashionable	2.75(1.82)	0.25	
	11. Smoking is good for social networking	4.87(1.86)	0.25	
	12. The life of a smoker is happier than that of a nonsmoker	3.03(1.85)	0.12	
**Perceived efficacy**	**Self-efficacy**			0.75
	13. I am confident that I can quit smoking successfully	4.32(2.11)	0.28	
	14. I have the ability to stop smoking	4.46(2.09)	0.27	
	15. I think stop smoking is easy for me	3.53(2.13)	0.13	
	**Response efficacy**			
	16. People will feel good by not smoking	4.31(1.92)	0.28	
	17. People will be less likely to get disease if they do not smoke	4.85(1.89)	0.38	
	18. Quitting smoking is good for disease recovery	5.43(1.81)	0.26	
**Perceived costs**	**Response cost**			0.73
	19. A person may be isolated if they quit smoking	3.34(1.92)	0.24	
	20. Refusing a cigarette offer is very impolite	3.85(2.03)	0.20	
	21. One will miss the enjoyment if he or she quits smoking	3.43(2.00)	0.17	
**Overall**				0.71

*ICC* represents the interclass correlation coefficient

### SEM results

**Fig. 2:**
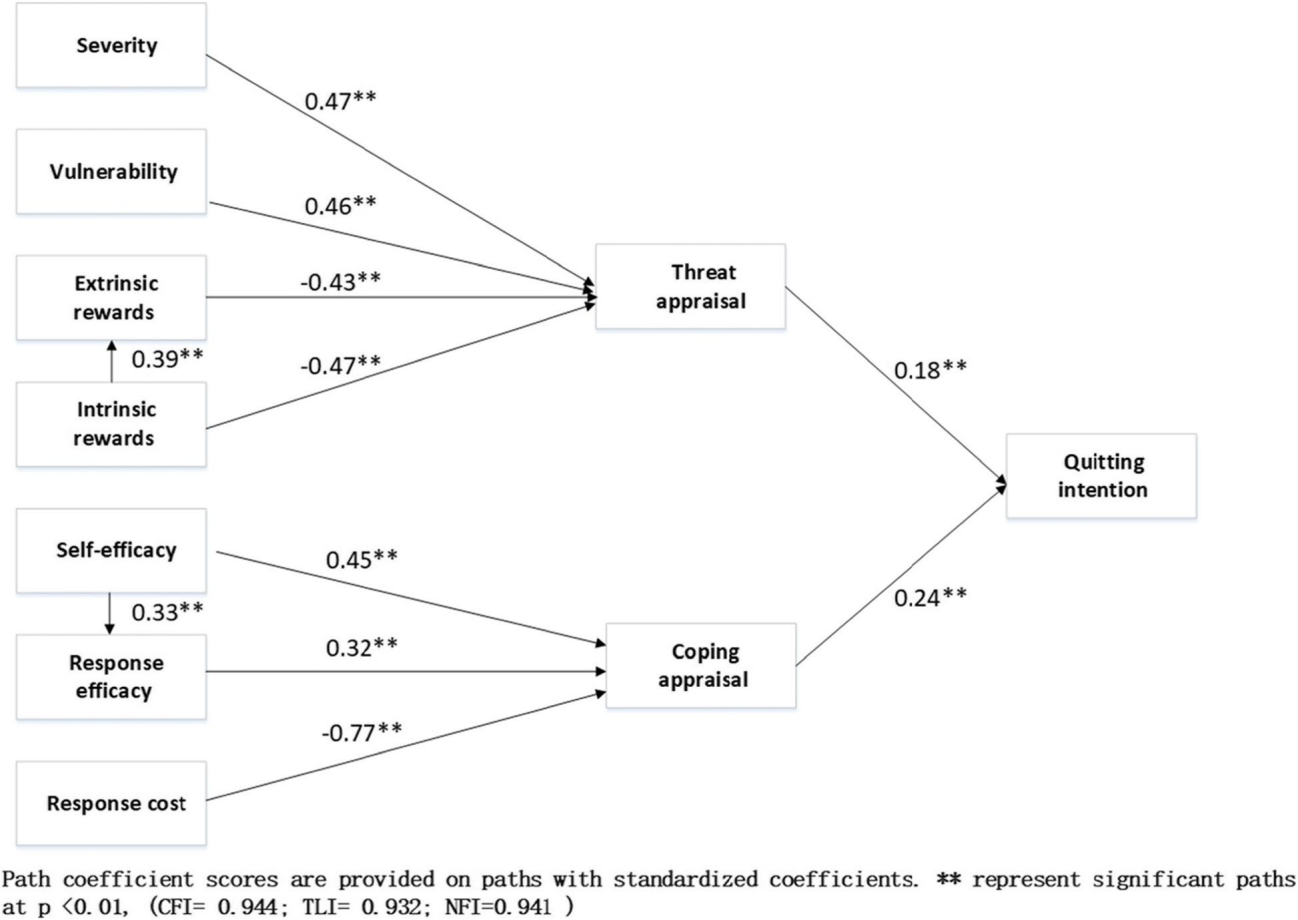
Standardized coefficients of the pathways from PMT constructs to quitting intention

Figure 2 shows the standardized coefficients of the pathways from PMT constructs to quitting intention. Greater intentions were significantly associated with higher threat appraisal (Coef. = 0.18, *P* < 0.01) and coping appraisal (Coef. = 0.24, *P* < 0.01). Threat appraisal was significantly associated with higher perceived severity (Coef. = 0.47, *P* < 0.01) and vulnerability (Coef. = 0.46, *P* < 0.01) but inversely associated with extrinsic rewards (Coef. = -0.43, *P* < 0.01) and intrinsic rewards (Coef. = -0.47, *P* < 0.01). Coping appraisal was significantly associated with higher self-efficacy (Coef. = 0.45, *P* < 0.01) and response efficacy (Coef. = 0.32, *P* < 0.01) but inversely associated with response cost (Coef. = -0.77, *P* < 0.01). The fit of this model was acceptable (CFI: 0.97; TLI: 0.95; NFI: 0.97; RMSEA: 0.06). The R^2^ of quitting intention was 0.12, which means that 12% of quitting intention was predicted by PMT constructs.

### Mediation analysis

Figure 2 shows that extrinsic rewards may be a potential mediator in the mechanism between intrinsic rewards and threat appraisal and that response efficacy may be another potential mediator in the mechanism between self-efficacy and coping appraisal. We then performed bootstrap tests to estimate how much of the effect is mediated through the channel of extrinsic rewards and response efficacy. The results for bootstrap tests are reported in Table 3. Extrinsic rewards and response efficacy were mediating factors. For threat appraisal, approximately 19.8% of the effects on lower threat appraisal were mediated by higher extrinsic rewards. For coping appraisal, approximately 42.8% of the effects on higher coping appraisal are mediated by higher response efficacy.

**Table 3:** Bootstrap test of mediation

Bootstrap test	Total effect	Direct effect	Indirect effect	Proportion of total effect that is mediated
**Mediating effect of response efficacy**				
Model 1: Self-efficacy → Coping appraisal	0.626**	0.502**	0.124**	19.80%
Model 2: Self-efficacy → Coping appraisal	0.630**	0.505**	0.125**	19.84%
**Mediating effect of extrinsic rewards**				
Model 1: Intrinsic rewards → Threat appraisal	-0.480**	-0.277**	-0.203**	42.29%
Model 2: Intrinsic rewards → Threat appraisal	-0.476**	-0.272**	-0.204**	42.86%

^**^p < 0.01

Model 1-we only included the independent variable, dependent variable and mediating variable in the model

Model 2-In addition to the independent, dependent and mediating variables, we also included control variables (age, sex and life satisfaction) in the model

## Discussion

This study contributes to the ongoing debate about factors associated with quitting intention and provides evidence from a more general perspective. Using PMT, this study provides a comprehensive picture of the possible factors related to quitting behaviour and how these influencing factors interact with each other. This study provides new and potentially important information. It successfully shows that PMT is a sound theoretical framework for predicting quitting intention among adults (first hypothesis). Despite substantial tobacco control efforts in China, studies have found limited success in reducing smoking prevalence. According to the Chinese Adult Tobacco Survey, the smoking prevalence for males was 52.9% in 2010 [27], 52.1% in 2015 [28] and 50.5% in 2018 [21]. One of the most important reasons for these high prevalences is the lack of relevant scales to measure the essential constructs in the theoretical approach. Due to this challenge, most tobacco control programmes are not grounded or guided by theory. Our study has proven the acceptability and reliability of the application of PMT, a Western-developed theory for behavioural change, in Asian countries. PMT can be used to assess quitting intention in adults. This is a critical step towards effective smoking cessation intervention.

Regarding the second hypothesis, based on the coefficients of PMT pathways, we found that coping appraisal has a stronger effect than threat appraisal. The pathway of coping appraisal assesses a person’s ability to deal with a threat and to reject it. An increase in coping appraisal increases the will to and results in the possibility of showing adaptive behaviour. Most tobacco control efforts focus only on educating tobacco harm and benefits for quitting, while coping skills are always ignored. Our results are consistent with previous research suggesting that coping appraisal is a better predictor of health behaviour than threat appraisal [17]. Therefore, our study may challenge ongoing tobacco control programmes if they assist people in identifying the potential benefits of quitting or harm of smoking (threat communication) as the only priority.

When all the PMT measures were analysed, most PMT components were significantly related to quitting intention, but the strength of the relationships differed for different PMT constructs. For example, response cost had the strongest effect. This is different from other Western-based research results and raises the question of why response cost could act as the strongest predictor of all the variables. A possible explanation is that smoking is not only a habit but also a part of traditional cultural and a part of social intercourse in China. China has a very long history of smoking, similar to tea culture, which is deeply rooted in people’s daily lives. In addition, the role of tobacco in social currency has been promoted by the tobacco industry, which has added traditional values and cultural customs to smoking behaviour to make smoking acceptable and desirable [27]. Therefore, when people think about stopping smoking, the response cost is far higher than simply quitting a bad habit.

Regarding the last hypothesis, we did indeed find two mediators: extrinsic rewards and response efficacy. The change in quitting intention reflects a synergism of multiple factors, with direct effects and indirect effects working in conjunction to influence quitting intention. Our results suggest that higher intrinsic rewards are associated with an increase in levels of extrinsic rewards, and these increases are subsequently associated with a significant decrease in threat appraisal. The largest mediating effect was found for response efficacy; such a mediating role between self-efficacy and coping appraisal can explain 42.8% of the effect of self-efficacy. This suggests that adults’ high confidence in their ability to quit is associated with a strong belief about the effectiveness of the quitting behaviour, which is in turn related to behaviour.

As an impressive finding, our data suggested that this emphasis on some PMT constructs due to the relatively low subscale score for individuals does not mean that we should ignore other constructs to achieve synergetic effects. For example, if someone’s threat appraisal is weak due to a very low score for extrinsic rewards, interventions designed only for this factor are not enough. To trigger stronger quitting intentions or maximize the impact of threat appraisal, interventions should at least influence intrinsic rewards because extrinsic rewards serve as the channel of the mediating effect from intrinsic reward to threat appraisal.

Our study has several limitations. First, we only used cross-sectional data for this estimation. As a result, we cannot infer a causal relationship. Second, most of our participants were male. Clearly, the inclusion of females might have led to different results. Third, all the information we collected was self-reported without verification, and respondents’ definitions of some questions may have varied. Fourth, the results could be biased because they are not based on nationally representative data. However, as the participants were recruited from different provinces of China, it is believed that the overall findings are meaningful. Fifth, we did not test interrater reliability, which may cause bias since there were many interviewers involved in the data collection.

Despite these limitations, our findings have practical implications and potential theoretical interest. First, this is the first research study focusing on the application of PMT, a Western-developed theory, in examining quitting intention among adults. This study provides new evidence supporting the utility of behavioural change theories developed in the West to promote smoking cessation efforts in China and other Asian countries. Second, we improved and assessed a measurement scale for adults’ quitting intention based on the existing PMT scale, which provides a tool for assessing the social cognitive processes underlying quitting behaviour. This is essential for more effective smoking cessation interventions. Third, future studies need to explore other potential mediators between extrinsic rewards and intrinsic rewards and other possible factors in the pathway between perceived factors and quitting intention. For example, the channel of extrinsic rewards only mediated a maximum of 19.8% of the total effects. What are the other parts of the mechanism? How do they contribute to a persistent enhancement of quitting intention? These issues have strong policy implications but are not easy to answer.

Based on the findings of this study, we can make specific policy recommendations. First, health practitioners should be encouraged to conduct evidence-based health interventions. Policy makers and scholars should develop tools, such as PMT scales, that can assess intention and related factors to support community health practitioners in designing effective intervention activities. Second, not all PMT factors have the same strength in predicting intention; health interventions should be directed at more influential PMT constructs, especially the factors that can have direct and indirect impacts on target intention and behaviour.

## Conclusion

This study finds that PMT is a sound theoretical framework for predicting quitting intention among adults. Coping appraisal has a stronger effect than threat appraisal for predicting quitting intention. Among the seven basic PMT constructs, the response cost has a more significant effect than the others. Mediation analyses confirmed that extrinsic rewards and response efficacy mediated the relationship between PMT constructs and quitting intention. Our findings are essential for understanding quitting behaviour among adults and support more effective smoking cessation activities. Taken together, the results of this empirical analysis not only contribute to identifying the determinants of quitting intention among adults but also provide further evidence regarding smoking behaviour in a developing country to add to earlier research on this topic.

## Data Availability

The data of the studies are accessible via Peking University, School of Public Health. Please contact the corresponding author.
